# Shared decision making and decision aids in the management of kidney disease and renal replacement treatment options

**DOI:** 10.1097/MNH.0000000000001140

**Published:** 2025-11-21

**Authors:** Yaara Zisman-Ilani, Connie M. Rhee, Fawaz Al Ammary, Kamyar Kalantar-Zadeh

**Affiliations:** a Department of Social and Behavioral Sciences, Barnett College of Public Health; b Department of Psychiatry and Behavioral Science, Lewis Katz School of Medicine, Temple University, Philadelphia, PA, USA; c Department of Clinical, Educational and Health Psychology, Division of Psychology and Language Sciences, University College London, London, UK; d Division of Nephrology, David Geffen School of Medicine at UCLA; e Nephrology Section, Veterans Affairs Greater Los Angeles Healthcare System, Los Angeles; f Department of Medicine, University of California Irvine, Orange; g Division of Nephrology, Lundquist Institute at Harbor-UCLA Medical Center, Torrance; h Division of Nephrology, Hypertension, and Kidney Transplantation, Department of Medicine, University of California Irvine School of Medicine, Orange, CA, USA

**Keywords:** chronic kidney disease, decision aids, decision support tools, shared decision making

## Abstract

**Purpose of review:**

To examine recent developments in shared decision making (SDM) interventions for advanced chronic kidney disease (CKD) and end-stage kidney disease (ESKD). Given the complexity of treatment decisions and low patient engagement despite available options, SDM is a critical approach to improve treatment initiation and engagement.

**Recent findings:**

Three recent SDM interventions were identified: DART (Decision-Aid for Renal Therapy), a decision support patient-centered video tool for older patients with stages 4–5 CKD that significantly reduced decisional conflict and improved treatment knowledge; YoDCA (Yorkshire Dialysis and Conservative Care Aid), a 28-page patient-centered decision support tool supporting dialysis versus conservative management decisions; and SIMPLIFY-HD (Stroke-Prevention Strategies in Patients With Atrial Fibrillation Receiving Maintenance Hemodialysis), an encounter-based decision aid for patient-provider use. These interventions demonstrated improved decision quality, reduced decisional conflict, and enhanced patient knowledge.

**Summary:**

While recent advances show promise for enhancing patient knowledge and decision-making among older adults with kidney disease, significant gaps remain. Limited real-world testing, narrow focus on older populations with late-stage disease, and insufficient integration of multimorbidity present implementation challenges. Future research should prioritize rigorous randomized controlled trials, broader patient inclusion, multimorbidity integration, clinician training, and assessment of long-term clinical outcomes to achieve patient-centered kidney care.

## INTRODUCTION

Chronic kidney disease (CKD) is one of the most common chronic conditions, affecting approximately one in seven adults, or 15% of the adult population in the United States [1,2]. Most individuals with CKD experience multimorbidity, commonly with conditions such as hypertension, diabetes mellitus, and cardiovascular disease [3–7], making the management of CKD significantly complex for patients, families, and healthcare providers [8]. Optimal management of CKD is critical to preventing disease progression and the development of end-stage kidney disease (ESKD), an advanced stage of CKD, which requires kidney replacement therapy (i.e., dialysis or a kidney transplant) for survival. While intended to be life-saving, dialysis is associated with increased mortality [9], improved physical function, reduced quality of life [10–12], and heightened risk of depression, anxiety, and dialysis withdrawal [13–17].

In contrast to some other chronic conditions, CKD presents patients, care partners, and providers with a broad range of treatment options. Depending on the stage of CKD, these options may include medications, lifestyle modifications, or even watchful waiting with no immediate intervention. Decisions may involve initiating dialysis, choosing the dialysis modality (in-center hemodialysis versus home-based peritoneal dialysis or home hemodialysis), pursuing a kidney transplant, whether preemptively (before dialysis initiation) or on dialysis, or managing co-existing comorbidities (i.e., diabetes, cardiovascular disease) in patients receiving dialysis [18]. Yet, despite the ESKD risks of health deterioration and mortality, the utilization of and engagement with these treatment options among CKD patients remains low [19–22].

Continuous patient involvement in treatment planning from the outset is critical for initiating and sustaining engagement in kidney care [18,23]. When patients are knowledgeable about their condition and treatment options, they are more actively involved in decision-making and, therefore, more likely to follow through with treatment [24,25]. However, meaningful involvement requires more than just providing medical information; it also requires patient-centered discussions that align with each patient's values, preferences, and circumstances. For example, offering living kidney donation without considering a patient's cultural context may feel alienating for the patient and the potential donor, which might have contributed to declines in living kidney donations among biologically related donors over the last two decades [26,27]. Mismatch between patient preferences and treatment options can reduce adherence and erode patient trust in provider, which ultimately affects treatment adherence. Building trust through open conversations and supporting patients in managing their care by setting clear expectations and reinforcing self-efficacy is essential for treatment initiation and long-term patient engagement. 

**Box 1:**
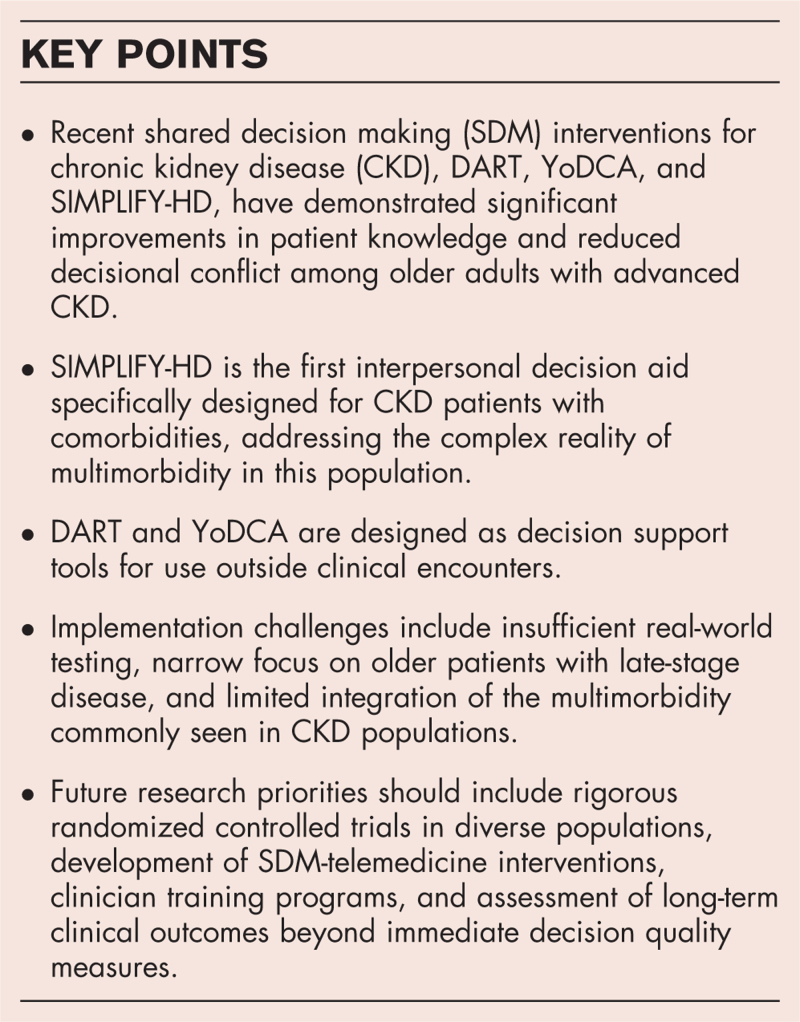
no caption available

**FIGURE 1:**
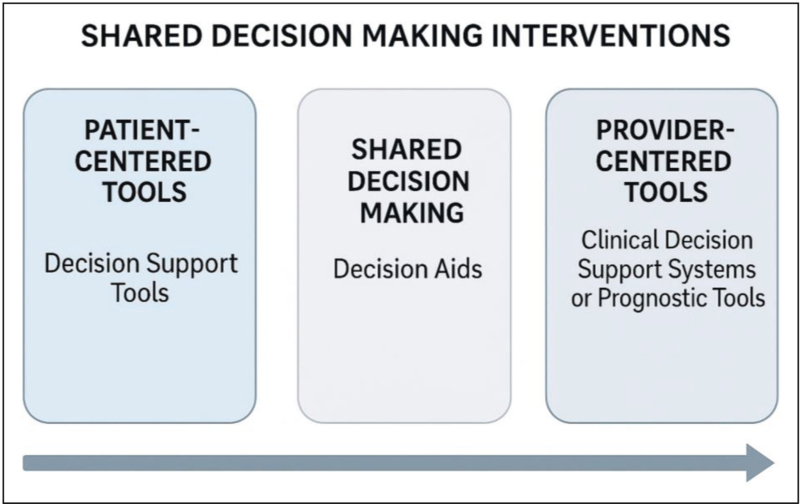
A spectrum of shared decision making interventions: from patient-centered tools to provider-centered tools.

Shared decision making (SDM) is a health communication approach designed to improve the quality of medical decision-making and enhance treatment initiation and engagement [28,29]. SDM aims to address the challenges outlined above within the context of medical care, which are often characterized by time constraints, information overload, and the need to make critical health decisions involving multiple parties, including patients, providers, and sometimes care partners, who may have differing levels of knowledge, expectations, motivations, or communication styles [30]. SDM interventions can be grouped into three main categories positioned along a spectrum (Fig. 1). On one end of the spectrum are patient-centered tools, also known as decision support tools, which help patients clarify their values and understand treatment options outside of an appointment. On the other end of the spectrum are provider-centered tools, including clinical decision support or prognostic tools, designed to support clinicians in interpreting data and making evidence-informed recommendations. In between these approaches are decision aids, which focus on enhancing interpersonal communication between patients and providers to improve real-time decision-making during the appointment [31]. While decision support tools (for patients) and clinical decision support tools (for providers) are often used outside the clinical encounter to prepare for decision-making, decision aids are typically used during appointments. Decision aids are often introduced by the provider to help the patient understand the condition and explore treatment options. In some cases, decision aids also assist providers, especially when the decision area lies outside their usual expertise. Decision aids have been shown not only to improve patient knowledge (similar to other support tools), but also to enhance self-efficacy in decision-making, health literacy, trust in providers, and a sense of involvement and ownership in the decision-making process, which is often associated with treatment engagement over time [32].

SDM interventions can be a natural fit in kidney care given the many treatment options to choose from [33]. However, the availability of evidence-based kidney SDM tools and interventions is limited, and the implementation of existing SDMs has been slow [34,35] and lags behind SDMs developed for other chronic conditions, such as hypertension [36], diabetes mellitus [37,38], or psychiatric conditions [37,39–41]. Although kidney disease commonly co-occurs with hypertension and diabetes, existing hypertension and diabetes mellitus SDM tools are not appropriate for kidney patients, as they were primarily designed to address a single condition, diabetes or hypertension, and do not account for the complexity of comorbid CKD and ESKD. The purpose of this literature review is to present recent developments in effective SDM tools and decision aids for CKD management, with particular emphasis on implementation science insights and the translation of research findings into clinical practice.

## RECENT ADVANCES IN SDM TOOLS FOR KIDNEY DISEASE MANAGEMENT

Recent reviews demonstrate a growing adoption of decision support tools for the management of CKD and ESKD [18,30,33]. These tools lie on one end of the SDM continuum, providing information without direct healthcare provider involvement [42]. Designed for independent patient use outside clinical consultations, these tools help patients and their care partners better understand their condition and treatment options while preparing them for appointments by addressing the foundational SDM component of information sharing [28,29], thereby potentially allowing the clinical encounter to focus more directly on decision-making within the constraints of limited appointment time. Decision support tools offer several additional advantages: they improve health literacy regarding CKD, ESKD, and treatment options, are accessible for patients as they can be used anytime and anywhere, and facilitate easy implementation without requiring integration into routine clinical workflows since they operate outside the clinical encounter.

Nevertheless, decision support tools have inherent limitations that limit their effectiveness within the SDM framework. Although these tools may utilize digital formats such as apps or videos, they typically lack interactivity, precluding real-time discussion and mutual patient-provider feedback. The absence of healthcare providers during the deliberation process means that critical SDM components, including clarification of patient values and preferences, collaborative deliberation, and provider guidance, are often missing. Consequently, the decision-making burden shifts disproportionately to patients, potentially diminishing SDM's overall impact. Furthermore, the information presented in these tools tends to be generic rather than personalized to individual patient needs, backgrounds, or clinical presentations. There is also considerable variability in the quality of decision support tools, as not all tools adhere to International Patient Decision Aid Standards (IPDAS) guidelines, which mandate clear presentation of treatment pros and cons alongside established quality criteria. Ultimately, many decision support tools emphasize the informational dimension of SDM and health literacy improvement while neglecting the interactive and deliberative elements that constitute authentic shared decision-making [43].

We identified two decision support tools for advanced CKD and ESKD since 2022 (Table 1). One is the DART (Decision-Aid for Renal Therapy) [44^▪▪^]. DART is a 1-h interactive video designed according to IPDAS principles to educate older patients with stages 4–5 nondialysis dependent (NDD)-CKD about treatment options for kidney failure, including peritoneal dialysis, hemodialysis, home hemodialysis, kidney transplant, and medical management. DART was given to the patient prior to an upcoming appointment and was evaluated against usual care in a randomized trial involving 363 patients aged 70 and older with stages 4–5 NDD-CKD. The DART decision support tool led to significantly lower decisional conflict scores, which indicates greater satisfaction with decisions made for participants receiving the DART, and improved participants’ knowledge of treatment options at 3- and 6-month follow-ups. These benefits persisted overall at 18 months. Participants in the DART intervention group were also more likely to form clear treatment preferences, reducing the proportion of “unsure” patients from 58% to 28% at 3 months, compared with a decrease from 51% to 38% in the usual care group [44^▪▪^]. The study supports DART's feasibility in CKD care for older adults and shows its effectiveness in improving decision quality and clarity among older adults facing kidney replacement therapy decisions.

**Table 1:** Characteristics of recent SDM tools for patients with CKD

Study (author, year)	SDM type	Country	Study aim	Format	Intervention description	Study design and sample	Key outcomes
Decision aids in development							
Winterbottom *et al.* 2025	Patient decision support tool	UK	Development of a decision support tool, Yorkshire Dialysis and Conservative Care Aid (YoDCA), for adults aged >70 y with advanced CKD (stages 4–5), based on IPDAS criteria and endorsed by NICE	Booklet	The booklet aims to help determine which treatment option (dialysis or conservative kidney management) suits them best in the context of fitting in with their lifestyle, and support discussions with health professionals about transitions in kidney failure management, end-of-life care preferences, and treatment decisions that consider people's lifestyle, values, and individual medical history. The booklet is for use as part of routine kidney care education about treatment options, or independently with people with advanced CKD, families, and/or carers. https://www.kidneyresearchyorkshire.org.uk/yorkshire-dialysis-and-conservative-care-decision-aid/	Qualitative, user-centered design with 11 stakeholders	Not applicable
Decision aids with evidence of feasibility and/or effectiveness							
Ladin *et al.* 2023	Patient decision support tool (used before visit)	USA	Testing decision support tool for Renal Therapy (DART) for adults aged >70 y with advanced CKD	Web-based	An hour-long interactive video providing patient education on treatment options for kidney failure (or end-stage renal disease), including: peritoneal dialysis, hemodialysis, home hemodialysis, kidney transplant, and medical management. Target audience are patients. https://patient.health-ce.wolterskluwer.com/DART/programs	Multisite randomized clinical trial (*n* = 400, but 363 were randomized); 18-month follow-up	Reduced decisional conflict at 3 and 6 months; increased CKD knowledge at 3, 6, and 12 months
Massé *et al.* 2025	Decision aid (used during visit)	Canada	Development and field testing a decision aid for stroke prevention in adult patients with atrial fibrillation receiving hemodialysis	Web-based	A decision aid that includes information on the diagnosis, available treatment options, discussion of harms and benefits of each options, deliberation of patient preferences, and decision-making in partnership. Designed for use as decision support tool or as a decision aid.	Quasi-experimental; 16 patients and 10 clinicians	Mean consultation length 21 (SD, 8) min; reduced decisional conflict; improved knowledge

CKD, chronic kidney disease; SDM, shared decision making.

The second decision support tool is the YoDCA (Yorkshire Dialysis and Conservative Care Aid) [45^▪▪^]. Although YoDCA has not yet been tested for feasibility or effectiveness, similar to the DART, the YoDCA was designed to support older patients with stages 4–5 NDD-CKD before an appointment to evaluate dialysis vs. conservative management. The YoDCA is a 28-page booklet with prompts and explanations. While YoDCA requires further rigorous evaluation, initial qualitative feedback as part of the development stages suggests that YoDCA has the potential to enhance patient understanding and communication around these two treatment options before they meet with their provider.

We identified one decision aid, the SIMPLIFY-HD (Strategies in Patients With Atrial Fibrillation Receiving Maintenance Hemodialysis) [46^▪▪^]. In a three-phase mixed-methods study including literature review, interviews with patients and clinicians for further refinement and a before-and-after quasi-experimental pilot, the IPDAS-informed SIMPLIFY-HD was tested in a sample of 16 older adult patients with ESKD on hemodialysis and comorbid atrial fibrillation, as well as 10 providers. The pilot involved one-on-one simulated SDM discussions (not real clinical appointments) to explore stroke-reduction strategies: warfarin, apixaban, rivaroxaban, vs, no treatment. As with the other two studies of decision support tools [44^▪▪^,^45^^▪▪^], the main outcomes assessed in phase three of the pilot testing were reduction in decision conflict scores and improvement in patient knowledge postconsultation. Clinical consultations lasted an average of 21 min (SD = 8). The mean Decisional Conflict Scale (DCS) score was significantly lower after using the decision aid (41.0 vs. 13.6, *P* < 0.001) representing better decision satisfaction postappointment. The SIMPLIFY-HD also reduced the proportion of patients with high decisional conflict (81% vs. 19%, *P* = 0.002) and significantly improved the mean patient knowledge score (62.7 vs. 76.6, *P* = 0.001). Although this pilot did not include a control group, the investigators reported that the visit duration was considerably shorter than the average time required to evaluate stroke-prevention strategies in atrial fibrillation.

The SIMPLIFY-HD represents an important advancement in both the SDM and ESKD fields. First, it addresses the common comorbidity of ESKD with other related chronic conditions, in this case atrial fibrillation. Given the high prevalence of chronic comorbidities among patients with ESKD, this complexity must be rigorously addressed as part of routine clinical care. However, most existing SDM tools and interventions are designed to address a single chronic condition, limiting their face validity and applicability to real-world situations where patients manage multiple chronic conditions. Therefore, the implementation potential of SIMPLIFY-HD in routine ESKD care is high. SIMPLIFY-HD also involves the use of an interpersonal SDM tool, a decision aid, between a patient and a provider during appointments, representing an important advancement for SDM in ESKD management. However, it has been tested only in controlled simulations, not in real clinical encounters or via randomized controlled trials, and with a small sample size. Thus, further rigorous evaluation is required to determine its effectiveness.

## IMPLEMENTATION OPPORTUNITIES AND CHALLENGES FOR SDM IN CHRONIC KIDNEY DISEASE

Recent research in CKD and SDM shows promising results, particularly in terms of acceptability and knowledge gains. The DART and SIMPLIFY-HD studies demonstrated measurable improvements in decision quality and patient knowledge, supporting their potential usability in CKD care. These tools were specifically tailored to the needs of older adults with advanced CKD, suggesting their potential scalability in this population. Importantly, they address a gap in SDM research, as older adults are often excluded from SDM studies across conditions due to concerns about cognitive impairments potentially affecting decision-making capacity [47–50]. These studies also advance SDM in the context of multimorbidity. For example, SIMPLIFY-HD addresses co-existing conditions, such as atrial fibrillation, offering a more realistic and patient-centered approach for individuals with complex health needs. The implementation potential of these tools is high: SIMPLIFY-HD reduced the average duration of decision-making consultations in simulated settings, and DART, as a video-based patient-facing decision support tool, is inherently easier to implement in routine care. Both tools were developed in accordance with IPDAS, increasing their likelihood of acceptance and adoption in clinical practice and supporting broader standardization of CKD-related SDM interventions.

However, several challenges remain. First, there is a need for increased real-world testing of SDM tools. While promising, neither DART nor SIMPLIFY-HD has been fully evaluated in actual clinical settings or integrated into routine care workflows. SIMPLIFY-HD, for example, was only tested in simulations, and the YoDCA tool has yet to undergo formal feasibility or effectiveness evaluation. To enhance the rigor of future studies, larger and more diverse samples are needed, as well as the inclusion of a control arm and real-world care settings implementation assessments. Current research is also limited to older adults with advanced CKD, excluding younger individuals and those at earlier stages of disease progression. Second, the DART and the YoDCA do not address the high prevalence of comorbidities in CKD populations. SIMPLIFY-HD is a notable exception, as it incorporates considerations for patients with both CKD and atrial fibrillation. However, the majority of SDM tools in general remain condition-specific and may not reflect the complex clinical realities encountered by providers or the multifaceted decision-making needs of patients. For instance, glucose monitoring in patients with both diabetes and CKD poses unique challenges. Although tools and clinical guidelines exist for continuous glucose monitoring in type 1 and type 2 diabetes, they have not been evaluated in populations with both diabetes and CKD [51].

## A PATH FORWARD

Recent advances in SDM for CKD highlight the promise of decision support tools and decision aids to enhance patient knowledge, clarify preferences, and improve decision quality among older adults with advanced CKD. Yet critical gaps persist, including limited real-world testing, underrepresentation of younger patients and those at earlier stages of CKD, and insufficient integration of multimorbidity into SDM frameworks. Addressing these gaps requires rigorous, practice-based evaluations and broader inclusion of diverse patient populations [31,52,53]. As decision support tools proliferate, future work should focus on developing SDM-telemedicine interventions designed for real-time consultations, particularly in telehealth settings where dialysis patients and providers are often in different locations [54]. Clinician training in SDM and patient-centered communication must be prioritized and integrated into medical school curricula and later reinforced during fellowship training programs [55]. Current SDM studies in CKD have primarily focused on immediate outcomes such as reductions in decisional conflict and improvements in patient knowledge. However, evaluating long-term clinical outcomes such as survival, treatment adherence, and quality of life, is essential for broader support and investment in SDM from healthcare systems and policymakers.

## CONCLUSION

Progress has been made in developing and evaluating SDM tools for older adults with CKD, with evidence of improved decision quality. However, rigorous real-world evaluation across broader patient populations remains limited, posing challenges to widespread implementation. Future research should prioritize rigorous evolutions via randomized controlled trials, integration of multimorbidity, inclusion of diverse populations, SDM-telemedicine adaptations for dialysis settings, clinician training, and assessment of long-term clinical outcomes. As SDM advances toward becoming a standard of care, coordinated efforts among researchers, clinicians, and patients are vital to achieving truly patient-centered, value-based kidney care.

## Acknowledgements

*None.*

### Financial support and sponsorship

*Zisman-Ilani – none; Rhee – supported by the National Institute of Diabetes and Digestive and Kidney Diseases (NIDDK) under award numbers R01-DK124138, R01-DK132869, R01-DK132875, and R01-DK122767; Al Ammary – none; Kalantar-Zadeh – NIH NIDDK; VA ORD; Lundquist Institute at Harbor-UCLA.*

### Conflicts of interest

*There are no conflicts of interest.*

## REFERENCES AND RECOMMENDED READING

Papers of particular interest, published within the annual period of review, have been highlighted as:

▪ of special interest
▪▪ of outstanding interest
